# Factors associated with TB screening among agricultural workers in Limpopo Province, South Africa

**DOI:** 10.1080/16549716.2022.2162227

**Published:** 2023-01-20

**Authors:** Nosimilo Mlangeni, Molebogeng Malotle, Felix Made, Jonathan Ramodike, Yandisa Sikweyiya, Christine Du Preez, Nikki Stuart Thompson, Muzimkhulu Zungu

**Affiliations:** a National Institute for Occupational Health, A division of the National Health Laboratory Service, Johannesburg, South Africa; b Department of Global Health, Stellenbosch University, Cape Town, South Africa; c School of Health Systems and Public Health, University of Pretoria, Pretoria, South Africa; d Gender & Health Research Unit, South African Medical Research Council, Pretoria, South Africa; e School of Public Health, University of the Witwatersrand, Johannesburg, South Africa; f Hoedspruit Training Trust (Hlokomela), Hoedspruit, South Africa; g CHoiCe Trust, Tzaneen, South Africa

**Keywords:** Agricultural workers, occupational health, TB prevention, migrant health, access to healthcare, workers’ health

## Abstract

**Background:**

Tuberculosis (TB) continues to be a public health issue of concern in South Africa. Workers in the agricultural sector are generally at increased risk of TB due to multiple interacting factors such as exposure to silica dust, co-worker infection, and occupations falling within the lower socio-economic sectors.

**Objective:**

This study investigates factors associated with TB screening uptake for agricultural workers in Limpopo Province, South Africa.

**Method:**

This cross-sectional study targeted a study population of 16,787 agricultural workers across 96 agricultural worksites in South Africa. A two-stage cluster random sampling design identified 24 agricultural worksites and a potential 2500 participants. The outcome variable was self-reported TB screening. Descriptive statistics and unadjusted and adjusted logistic regression analyses were performed to determine factors associated with TB screening. A literature review informed the selection of covariates as possible confounders.

**Results:**

The final study sample comprised 2144 workers across 24 sites, with 55% being women. TB screening uptake was 1155 (56.3%). Factors such as living with human immunodeficiency virus (HIV) (AOR 3.16, 95% CI: 2.44–4.09), accessing health services in the workplace (AOR 1.94, 95% CI: 1.09–3.46), and having prior TB knowledge (AOR 18.45, 95% CI: 9.8–34.74) were positively associated with TB screening. Participants in the age group 36–49 years had significantly higher odds of self-reporting TB screening, compared with those aged 18–25 years (AOR 1.37, 95% CI 1.07–1.77). Migrant workers from Mozambique (OR 0.52, 95% CI: 0.34–0.79) and Zimbabwe (OR 0.71, 95% CI 0.57–0.89) were significantly less likely to self-report TB screening compared to their South African counterparts.

**Conclusion:**

The findings underscore the importance of workplace health services in achieving end-TB targets. We recommend programs and interventions for preventing TB in South Africa that target the agricultural sector in general, and in particular migrant workers.

## Introduction

Tuberculosis (TB) continues to be a global public health issue [1]. In 2020 the global TB incidence rate was 127/100,000 population and 9.9 million people were reported to have been ill with TB that same year [2]. South Africa is one of the countries with the highest TB burden, with an incidence rate of 554/100,000 population in 2020 [2]. Although South Africa has a well-established TB control program [3], the high TB incidence remains a public health concern [4]. In South Africa, TB is a leading cause of death among people living with the human immunodeficiency virus (HIV) [1] and a high burden of TB/HIV co-infection, at 394/100,000 population [2]. The main drivers of TB are HIV, migration, poverty, and social and economic disadvantages. Specific factors such as overcrowding, inadequate ventilation, and occupational risks also contribute [5,6].

The World Health Organization’s (WHO) Global TB Strategy targets include a 90% reduction in new TB cases and full TB elimination by 2050 [7]. Similarly, South Africa, through the National Strategic Plan for HIV, TB and STIs 2017–2022 [8], is committed to the 90-90-90 WHO TB targets [9]. The targets aim to have: at least 90% of the population screened for TB and placed on appropriate therapy; 90% of the most vulnerable populations screened for TB and given access to appropriate therapy, and achieved 90% of treatment success in all people diagnosed with TB. The WHO reports that most countries do not report national TB testing data, resulting in a dearth of global TB screening statistics [10]. TB statistics for most vulnerable populations are also lacking, except for people living with HIV (PLHIV), of whom 43% have been reached [10]. South Africa has achieved 80% of TB treatment success in people diagnosed with TB [10].

Some occupations have a higher risk of TB morbidity due to exposure to TB, silica dust, and occupations falling on the lower socio-economic status [11]. Agricultural work is a high-risk occupation, due to several socio-economic factors, such as poor living and working conditions, migration status, and challenges in accessing health services [6,12–14]. Exposure to silica in the agricultural industry has been reported in South Africa, and this poses risks for workers [15]. Agricultural workers are also a vulnerable population, as they work and live in hard-to-reach areas, and a high proportion are migrant workers [9]. Although there is a dearth of studies on TB incidence and prevalence among agricultural workers globally, it has been reported that there is an increased risk of TB for agricultural workers when compared to workers in other occupations [16,17]. TB incidence of 1 685 per 10,000 population and 12.11 per 10,000 population was reported among agricultural workers in Boland (South Africa) and Saudi Arabia respectively [17,18].

TB mortality by occupation in South Africa was reported at 14.6% among agricultural workers. The odds of dying from TB were 58% higher among agricultural labourers compared to those in other occupations [11]. In South Africa, high TB/HIV co-infection rates are an important contributory factor to increased TB risk in this population [19].

Access to health services is an important challenge for agricultural workers, as is the case for other migrant labourers [12,20]. Poor access to health services reduces the chances of prompt screening and early TB detection [12,20]. To strengthen TB screening in hard-to-reach populations, South Africa has implemented an active case-finding and contact tracing program, which is community-based and targets high TB burden areas [8]. TB screening plays a crucial role in preventing and controlling TB as screening improves early detection of TB infection, thus reducing transmission and improving outcomes for those with TB [21].

The WHO together with the International Labour Organization advocates that workplaces are an appropriate setting for TB prevention and control activities because the workplace serves as an accessible and convenient setting for all categories of employees [22]. It is estimated that, globally, only 15% of workers receive essential health and basic occupational health services, while many poor and informal economy workers lack occupational health services [23,24].

The study’s objective is to investigate factors associated with TB screening uptake for agricultural workers in Limpopo, South Africa. The findings will inform TB screening strategies, and thus contribute toward the WHO 90-90-90 TB targets, especially for vulnerable populations [9].

## Methods

### Study design, setting, and population

This study was part of a cross-sectional survey that was conducted as an evaluation study following HIV intervention programs for agricultural workers in the Limpopo Province, South Africa. The Limpopo Province lies on the north-eastern side of South Africa and shares a border with Zimbabwe, Botswana, and Mozambique [25]. Due to its geographic location, Limpopo serves as an entry point for many regional labour migrants [26]. The province is also a prime agricultural region, mainly producing fruits and vegetables, further attracting internal and external agricultural migrant workers [27,28]. The Limpopo Province has five districts, and this study focused on two, namely the Vhembe and Mopani districts. The two districts and participating agricultural worksites were conveniently selected as they were part of an ongoing HIV program provided by non-governmental organizations (NGOs), and the NGOs were able to organize access to the participating agricultural worksites. The HIV program rendered to agricultural workers includes HIV and TB screening, peer education, and prompt referral and/or initiation of treatment for workers diagnosed with HIV or TB.

The study population comprised 16,787 agricultural workers from 96 agricultural worksites. The worksites consisted of commercial farms varying in size from small (about 20 workers) to large (>900 workers).

### Sampling

**Figure 1.**
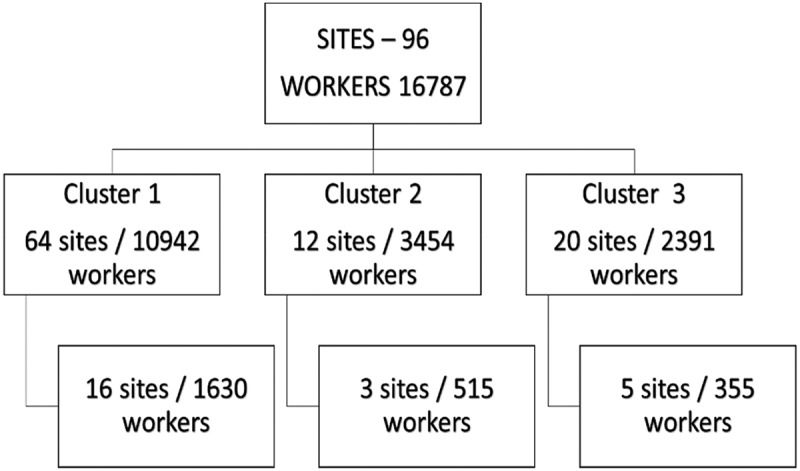
Sampling flow chart, showing the selection of participants.

The 96 agricultural worksites were grouped into three geographic clusters, under the three participating NGOs that serve them (Figure 1). Each NGO was considered a cluster, with one NGO in one district, and two NGOs in two different sub-districts of another district. A two-stage cluster sampling method was used. The first stage involved a sampling of agricultural worksites in each cluster. The total sample included 24 agricultural worksites comprising 16 agricultural worksites from cluster 1, three agricultural worksites from cluster 2, and five agricultural worksites from cluster 3. The second stage involved drawing the study sample. The effective sample size was 2101 (based on a 2% margin of error and a 95% confidence interval). We increased this to 2500, to allow for possible non-response. The sample size was divided by participating farms, taking farm size into account to achieve representation. A simple random sampling was conducted to select participating worksites. A systematic random sampling was conducted whereby every second worker was selected. All agricultural workers 18 years and older were eligible to participate in the study.

### Data collection and analysis

Pre-piloted, paper-based survey questionnaires were administered by trained data collectors who were all trained HIV counselors working for the NGOs running the HIV programs. The questionnaire was adapted from a baseline survey which was conducted as a pre-intervention study in the same study population [29]. Workers’ recruitment to the study was conducted after a presentation explaining the study on the day of data collection. The data collectors provided study-related information to potential participants and sought their informed consent. Participation was voluntary, with no monetary or non-monetary reimbursement.

Data were captured on Epi info version 3.5.1, and analysis was performed using both Epi info and STATA version 16. Workers were divided into three age groups, namely 19–25 years (reference category), 26–35, 36–49, and older than 49. Participants’ education level was classified as no education (reference category), primary, secondary, matric, and post-matric education. Nationality categories were determined according to previously reported nationalities in the study setting [29]. The outcome variable was self-reported TB screening. This was captured by asking participants if they had ever been screened for TB. Data were summarized using descriptive statistics, frequency distribution tables, and charts. A review of literature guided the selection of covariates [20,29,30]. Unadjusted logistic regression was performed to measure the association of TB screening with individual variables. Stepwise logistic regression, with robust estimation of standard errors to cater for the sampling design, was used to determine associations. Model building was done using the maximum likelihood ratio test, and all statistically significant variables (*p* < .05) in the univariate analysis were included in the final adjusted multivariate logistic regression model. The variables that were very close in predicting the TB screening remained in the final model. Results are presented as odds ratios (OR) with a 95% confidence interval (CI).

## Results

In total, 2500 workers were invited, and 2144 consented to participate, giving a response rate of 86%. More than half of workers 1181 (55.6%) were women, with 1567 (73.3%) being between the ages 26 and 49 years. Nine hundred and thirty-seven (43.8%) workers had attended secondary school, while 159 (7.4%) did not attend school at all. Most of the workers were South Africans (1594) (74.3%), while 431 (20.1%) were migrants from Zimbabwe. Most of the workers (1145) (53.6%) were employed on a part-time or seasonal basis (Table 1).

**Table 1.** Demographic and clinical characteristics of participants.

Characteristics	Frequency	Percentage (%)
Gender (n = 2,126)		
Male	945	44.4
Female	1,181	55.6
Age group (n = 2,138)		
19-25	291	13.6
26-35	787	36.8
36-49	780	36.5
>49	280	13.1
Education (n = 2,138)		
None	159	7.4
Primary	514	24.0
Secondary	937	43.8
Matric	474	22.2
Tertiary	54	2.5
Nationality (n = 2,139)		
South African	1,594	74.5
Mozambique	109	5.1
Swaziland	3	0.1
Zimbabwe	431	20.1
Other	2	0.1
Employment Status (n = 2,135)		
Full time	990	46.4
Part time	1,145	53.6
Living with HIV (n = 1,446)		
No	902	62.4
Yes	544	37.6
Ever heard about TB (n = 2,107)		
No	344	16.3
Yes	1,763	83.7
Ever screened for TB (n = 2,052)		
No	1,155	56.3
Yes	897	43.7
Lived with someone diagnosed with TB (n = 1,924)		
No	1,417	73.6
Yes	507	26.4
Worried about contracting TB (n = 2,010)		
Very worried	708	35.2
A little worried	360	17.9
Not worried at all	942	46.9
TB risk at workplace (n = 2,113)		
No	1,490	70.5
Yes	623	29.5

Of the 1446 (67%) participants who had been tested for HIV, 544 (37.6%) reported testing positive for HIV on their last test. About 344 (16.3%) participants had never heard of TB, while 1155 (56.3%) had never been screened for TB. A number of participants (507) (26.4%) reported having been in close contact with a family member who had TB. When asked whether they were worried about contracting TB, about 942 (46.9%) of participants were not worried at all, while 708 (35.2%) indicated that they were very worried. Most participants (1490) (70.5%) felt that their work did not put them at risk of contracting TB.

Table 2 indicates participants’ access to health services. Most of the workers (1895) (88.4%) indicated that they use government health services, while 147 (6.9%) workers said they could access health services provided by NGOs in their workplace, and 735 (34.3%) workers indicated that they had access to NGO health services outside the workplace. A large portion of workers (1447) (70.0%) indicated that they needed transport to get to the nearest primary health care (PHC) clinic, and 626 (32%) of the participants estimated the walking distance to the nearest PHC clinic to be more than 5 km. Twenty-nine percent of participants reported that they walk 2.1–5 km to the nearest PHC facility, and 39% reported a walking distance of 0–2 km to the nearest PHC facility.

**Table 2.** Accessible health services utilized by the workers.

Characteristics	Frequency	Percentage (%)
**Government health service (N = 2,144)**		
No	249	11.6
Yes	1,895	88.4
**NGO health services provided at the workplace (N = 2,144)**		
No	1997	93.1
Yes	147	6.9
**NGO health services outside the workplace (N = 2,144)**		
No	1,409	65.7
Yes	735	34.3
**Need transport to Clinic (N = 2,068)**		
No	621	30.0
Yes	1,447	70.0

### Factors associated with TB screening for agricultural workers

The unadjusted odds ratio (AOR) and the adjusted odds ratios for TB screening in relation to socio-demographics, access to health services, and TB awareness factors are shown in Table 3. Interestingly very few participants were able to access health services in their workplace. But of those who did, most were screened for TB. The unadjusted analyses revealed that workers who were able to access health services in their workplace were 2.53 times more likely to be screened for TB (OR 2.53, 95% CI 1.77–3.61), and a significant association was maintained on adjusted analysis (*p* = .025). Migrants who were from Zimbabwe (OR 0.71, 95% CI 0.57–0.89) and Mozambique (OR 0.52 95% CI 0.34–0.79) were less likely to be screened for TB; however, there was no significant association in the multivariate analysis.

**Table 3.** Univariate and multivariate logistic regression for factors associated with TB screening among agricultural workers.

Variable	n/N (%)	OR	p value	95% CI	AOR	P value	95% CI
Gender							
Male	385/911 (42.3)	1					
Female	502/1124 (44.7)	1.10	.278	0.92–1.32			
Age in years							
18-25	80/279 (28.7)	1					
26-35	304/756 (40.2)	1.67	.001	0.24–2.25			
36-49	387/746 (51.9)	2.68	.000	1.99–3.61	1.37	0.013	1.07–1.77
>49	123/266 (46.2)	2.14	.000	1.5–3.05			
Level of education							
None	63/149 (42.3)	1					
Primary	228/492 (46.3)	1.17	.383	0.81–1.70			
Secondary	386/895 (43.1)	1.03	.847	0.72–1.47			
High School	188/456 (41.2)	0.95	.821	0.65–1.39			
Tertiary	27/54 (50.0)	1.36	.329	0.73–2.54			
Nationality							
South African	701/1522 (46.1)	1					
Mozambique	32/104 (30.8)	0.52	.003	0.34–0.79			
Swaziland	1/3 (33.3)	0.58	.662	0.05–6.47			
Zimbabwe	158/417 (37.9)	0.71	.003	0.57–0.89			
Other	1/2 (50.0)	1.17	.911	0.07–18.75			
Years of work							
<3 years	355/903 (39.3)	1					
4–9 years	293/652 (44.9)	1.25	.027	1.02–1.54			
>10 years	237/470 (50.4)	1.57	.000	1.25–1.96			
Access to government service							
No	117/239 (49.0)	1					
Yes	780/1813 (43.0)	0.79	.083	0.6–1.03	0.54	0.004	0.35–0.82
Access NGO health services at workplace							
No	805/1910 (42.1)	1					
Yes	92/142 (64.8)	2.53	.000	1.77–3.61	1.94	0.025	1.09–3.46
Access NGO health services outside workplace							
No	607/1340 (45.3)	1					
Yes	290/712 (40.7)	0.83	.047	0.69–1.00	0.74	0.025	0.57–0.96
Need transport to clinic							
No	215/599 (35.9)	1					
Yes	657/1380 (47.6)	1.62	.000	1.33–1.98	1.64	0.000	1.25–2.16
Have tested HIV positive							
No	312/859 (36.3)	1					
Yes	315/516 (61.1)	2.75	.000	2.19–3.44	3.16	0.000	2.44–4.09
TB awareness							
No	14/312 (4.5)	1					
Yes	876/1727 (50.7)	21.91	.000	12.71–37.76	18.45	0.000	9.8–34.74
TB contact							
No	311/665 (46.8)	1					
Yes	149/349 (42.7)	1.69	.000	1.38–2.08			

The final multivariate model included gender, age in years, level of education, nationality, years of work, access to government health service, access to NGO health services in the workplace, need for transport to the clinic, having tested for HIV, TB awareness, and contact with a TB patient. The age groups 36–49 years were 1.37 times more likely to be screened for TB (AOR 1.37, 95% CI 1.07–1.77) as opposed to workers in the 18–25 years age group. The odds of accessing TB screening were 46% lower among agricultural workers reporting having access to government health services (AOR 0.54, 95% CI 0.35–0.82) and 26% lower among agricultural workers who could access NGO services outside the workplace (AOR 0.74, 95% CI 0.5–0.96). Having tested for HIV (*p* < .000) and being aware of TB (*p* < .000) were significantly associated with TB screening. Workers who needed transport to get to a health facility were 1.64 times more likely to be screened for TB (AOR 1.64, 95% CI 1.25–2.16). The AOR for TB screening among agricultural workers reporting being aware of TB was 18.45 times greater (AOR 18.45, 95% CI 9.8–34.74) (*p* < .000). All the variables included in the final multivariate model were significantly associated with TB screening.

## Discussion

This study was conducted to understand factors associated with TB screening among agricultural workers. Notably, fewer than half of agricultural workers reported being screened for TB. Factors that were positively associated with TB screening included awareness of TB, having a TB contact at home, being HIV positive, and having worked as an agricultural worker for four years or more. Workers were also more likely to be screened if they had TB services at their workplace, but very few workers had access to health services in the workplace.

The findings reveal that most agricultural workers could access public healthcare services; however, almost a third of workers (31.7%) lived beyond the 5 km radius of the nearest health facility. Living far from health facilities may have financial implications and may pose a barrier to accessing health services [31,32]. Agricultural workers who live far from health facilities need transport to get to the nearest health services, and many also need to take time off work to attend to their health needs. For most agricultural workers, especially temporal or seasonal workers, taking time off work to seek healthcare means a loss of income.

The study findings revealed low self-reported TB screening (43.7%) among agricultural workers. This low screening rate is below the 90% target of the end-TB WHO strategy [9]. When TB screening is low, there is reduced TB case finding, which makes prompt initiation of the correct treatment regimen highly unattainable for those who unknowingly live with TB [33]. One of the main concerns with vulnerable working populations, such as agricultural workers, is that they can easily be missed in the broader scope of the end-TB strategy. When the program outcomes are reviewed, without proper scrutiny of specific vulnerable population groups, it may appear that the program targets are being successfully reached. This is likely to provide misleading information about the attainment of program targets.

Previous studies have recommended strengthening outreach and awareness campaigns for agricultural workers [13,30,34] as a means of improving access and bringing prevention services closer to farming communities. The findings of this study support the call for targeted efforts in TB awareness and education, especially for hard-to-reach populations, including agricultural workers, who are unlikely to be reached through conventional methods. Notably, living with HIV was positively associated with TB screening. This highlights the positive outcomes of integrated HIV and TB programs, where patients who are HIV positive are screened for TB, and vice versa. Through this integration, HIV-infected patients know more about TB prevention and understand the importance of TB screening. NGOs thus play an important role in providing health services in our setting.

Having health services in the workplace increased the likelihood of screening for TB. Previous studies conducted in other countries have reported that migrant agricultural workers were hardly reached with health information, resulting in less knowledge about diseases [30,35]. Furthermore, a link between disease knowledge and screening uptake has long been established [36]. In this study, workers from Zimbabwe and Mozambique were less likely to have been screened for TB as compared to workers from South Africa. The reasons for this may be varied; migrant agricultural workers may have less awareness of TB, as has been the case in other studies [13,30]. Moreover, migrant agricultural workers are a highly mobile population who spend most of their time moving from one farming town to another, or between their country of origin and South Africa, in search of work.

It has been previously reported that gender and educational level influence the uptake of TB screening [13,37,38], with women and those with high school education more likely to screen. This was not the case in our study, as screening uptake was not associated with the level of education or gender. This finding suggests that structural factors, rather than individual factors, are the main barriers to TB screening in this population [39].

It is concerning that more than half of the workers in our study (64.8%) had a low perception of TB risk. Most workers were not aware of predisposing factors linked to their type of work. Studies have established a link between low-risk perception and inadequate use of preventive measures [36]. In the case of TB, screening is one of the preventive measures, leading to prompt initiation of a suitable treatment regime. Based on our findings, the low TB risk perception among agricultural workers in our study may explain the low TB screening uptake.

### Strengths and limitations

The major strengths of this study are the large sample size and good response rate. While we believe that our study sample provides a fair reflection of TB screening and access to healthcare in the study population, two districts were conveniently selected in one province of South Africa. This might limit the generalizability of the current findings to other parts of South Africa. High TB-related stigma and discrimination have been reported in past studies, which may affect screening uptake. In this study, we did not investigate whether stigma and discrimination played a role in TB screening uptake. Despite the limitations, we believe that this study provides a reasonable source of information for other researchers and policymakers.

## Conclusion

The findings highlight determinants of access to TB screening uptake among agricultural workers in Limpopo Province, South Africa. TB screening was more likely if workers had access to health services in the workplace, were non-migrant workers, and had tested positive for HIV. TB services for agricultural workers should ideally be situated in the workplace and be available to seasonal as well as permanent workers. Our findings indicate that NGOs play an important role in bringing health services to the workplace. We recommend reviews of policy and programmatic interventions on TB prevention and workplace health services in South Africa, particularly for agricultural workers and other hard-to-reach populations. Lastly, our findings provide the basis for further research to investigate access to TB programs for agricultural workers.

## Paper context

Agricultural workers are a vulnerable working population with poor access to health services. There is reportedly a high TB mortality rate within this population, but limited information on screening uptake. The results of this study indicate low TB screening, coupled with poor access to healthcare services. The study findings are important for planning TB prevention programs in the agricultural sector.
